# Purification and characterization of *Terfezia claveryi TcCAT-1*, a desert truffle catalase upregulated in mycorrhizal symbiosis

**DOI:** 10.1371/journal.pone.0219300

**Published:** 2019-07-10

**Authors:** José Eduardo Marqués-Gálvez, Asunción Morte, Alfonso Navarro-Ródenas, Francisco García-Carmona, Manuela Pérez-Gilabert

**Affiliations:** 1 Thader Biotechnology SL, Campus de Espinardo, Murcia, Spain; 2 Departamento de Biología Vegetal (Botánica), Facultad de Biología, Universidad de Murcia, Campus de Espinardo, Murcia, Spain; 3 Departamento de Bioquímica y Biología Molecular-A, Facultad de Biología, Universidad de Murcia, Campus de Espinardo, Murcia, Spain; University of Michigan, UNITED STATES

## Abstract

*Terfezia claveryi* Chatin is a mycorrhizal fungus that forms ectendomycorrhizal associations with plants of *Helianthemum* genus. Its appreciated edibility and drought resistance make this fungus a potential alternative crop in arid and semiarid areas of the Mediterranean region. In order to increase the knowledge about the biology of this fungus in terms of mycorrhiza formation and response to drought stress, a catalase from *T*. *claveryi* (TcCAT-1) has been purified to apparent homogeneity and biochemically characterized; in addition, the expression pattern of this enzyme during different stages of *T*. *claveryi* biological cycle and under drought stress conditions are reported. The results obtained, together with the phylogenetic analysis and homology modeling, indicate that TcCAT-1 is a homotetramer large subunit size monofunctional-heme catalase belonging to Clade 2. The highest expression of this enzyme occurs in mature mycorrhiza, revealing a possible role in mycorrhiza colonization, but it is not upregulated under drought stress. However, the H_2_O_2_ content of mycorrhizal plants submitted to drought stress is lower than in well watered treatments, suggesting that mycorrhization improves the plant’s oxidative stress response, although not via *TcCAT-1* upregulation.

## Introduction

Desert truffles are a group of edible hypogeous ascomycetes that form mycorrhizal symbiosis with plants of the *Cistaceae* family. Some species of *Terfezia*, *Tirmania* and *Picoa* belong to this group of truffles, which are distributed in arid and semi-arid areas, mainly around the Mediterranean countries, from the South of Europe to the North of Africa. *Terfezia claveryi* Chatin is one of the best known desert truffle species because of its great ecological and commercial value [1]. The type of association formed between this species and *Helianthemum* genus plants is an ectendomycorrhiza, characterized by the presence of both intercellular Hartig net and intracellular hyphae penetrating the cortex cells [2]. Furthermore, biotechnological advances have allowed cultivation and subsequent commercialization of this species using *Helianthemum almeriense* Pau as host plant [3,4].

Drought is one of the main limiting factors for photosynthesis, growth and survival of Mediterranean plants [5]. One of the consequences of hydric deficit in plants is oxidative stress, which implies an excessive accumulation of reactive oxygen species (ROS) such as superoxide anion and H_2_O_2_. Therefore, antioxidant systems, both enzymatic and non-enzymatic, must be involved in water stress tolerance mechanisms. It is well known that mycorrhizal fungi modify water relations in the host [6]. The majority of studies about this topic have been carried out in arbuscular mycorrhiza (AM) symbiosis and they suggest several mechanisms by which this symbiosis can alleviate drought stress in host plants [7]. One of these mechanisms is the protection against the oxidative damage generated by drought. In soybean inoculated with AM, there is a decrease in the oxidative damage to lipids in roots during water-deficit conditions [8]. Under drought stress, roots of AM *Digitaria eriantha* plants showed lower levels of H_2_O_2_ and higher catalase activity than their non-mycorrhizal counterparts [9]. Thus, fungal enzymes involved in the control of the levels of H_2_O_2_ may be involved in the enhanced resistance to drought stress of mycorrhizal plants. Mycorrhizal plants of *H*. *almeriense* with *T*. *claveryi* are well adapted to semiarid conditions and the negative effects of drought are reduced in these plants by specific physiological (transpiration, water use efficiency, aquaporin expression), nutritional (P, N and K content) and morphological alterations due to the mycorrhizal colonization [10–12].

It is well known that H_2_O_2_ at high concentrations is toxic to cells, but there are evidences involving this molecule in signaling, apoptosis or cell differentiation [13]. The intracellular colonization of the AM *Funneliformis mosseae* is known to be limited by the oxidative burst produced in *Medicago trunculata* host roots [14]. Baptista et al. [15], stated that during the early stages of ectomycorrhizal establishment of *Pisolithus tinctorious* x *Castanea sativa*, H_2_O_2_ accumulates following a pattern similar to the one showed by pathogenic fungi [16] and it is a candidate signaling molecule for symbiosis [17].

Catalases are the main enzymes responsible for the dismutation of H_2_O_2_ into water and dioxygen. Catalase activity has been observed in three groups of enzymes: “typical” monofunctional heme-catalases (the largest and most extensively studied), bifunctional catalases-peroxidases and non-heme catalases. In addition, low levels of catalase activity are found in many heme containing proteins not normally considered to be catalases [18]. Monofunctional heme-catalases have been classified following two criteria: subunit size and phylogeny. Based on subunit size they are divided in two groups: the large subunit size catalases, “LSCs”(>75 kDa), present only in bacteria and fungi and the small subunit size catalases “SSCs” (<60 kDa) [19, 20]. LSCs are classified in L1 and L2 subgroups. In fungi, L1-type catalases are not inducible and usually accumulate in spores, while those from the L2-type are usually induced by different stressors and are extracellular enzymes [19]. Both LSCs and SSCs have proved to be active as tetramers and dimers, but not as monomers [21]. On the other hand, typical catalases are divided in three main evolutionary clades: Clade 2, comprising LSCs and Clade 1 and 3 comprising SSCs. Clade 1 includes SSCs from bacteria, green algae and plants. Clade 2 groups the LSCs from bacteria and fungi while Clade 3 contains SSCs from most phyla, including fungi, but not Viridiplantae [19].

There is little knowledge about the physiological role of catalases in fungi, even less in mycorrhizal fungi, but catalases may be involved both in mycorrhiza formation and in defense against drought stress. Thus, in order to test the relevance of fungal catalases in these processes here we report on the purification and biochemical characterization of a catalase from *T*. *claveryi*, named TcCAT1, as well as its expression profile across different stages of *T*. *claveryi* life cycle and in *T*. *claveryi* mycorrhizal *Helianthemum* host plants under different water treatments.

## Materials and methods

### Biochemical and sequence analysis

#### Enzyme extraction and purification

Pieces of *T*. *claveryi* ascocarps collected in Zarzadilla de Totana (Lorca, Murcia, Spain) under *H*. *almeriense* shrubs, were homogenized using liquid nitrogen with a mortar and pestle and then suspended in 0.1 M sodium phosphate at pH 7.0 in a ratio of 1:4 (w/v). The homogenate was then centrifuged at 7,000 *g* for 20 min at 4°C in a Sigma 2-16K centrifuge with a 12141-H rotor (Sigma, Osterode am Harz, Germany). The resultant supernatant was subjected to temperature phase partitioning with a 10% (w/v) final concentration of Triton X-114 (TX-114) prepared in the same buffer. The mixture was placed in a thermostatic bath at 37°C for 30 min or until phase partitioning was achieved. This solution was centrifuged at 7,000 *g* for 20 min at 30°C. After centrifugation, the supernatant was either used immediately or stored at -80°C (where it was stable for more than one month). The buffer of the phase-partitioning supernatant was changed to buffer A (1.5 M ammonium sulphate in 10 mM Tris-HCl pH 6.9) by ultrafiltration using Amicon Ultra-4, 100,000 MWCO, (Merck Millipore Ltd., Ireland). Aliquots of 1 mL of this extract were loaded onto a HiTrap Phenyl HP 1 mL column (GE Healthcare Life Sciences, Barcelona, Spain) connected to an Äkta purifier (GE Healthcare Life Sciences, Barcelona, Spain) and equilibrated with buffer A at a flow rate of 0.75 mL.min^-1^. After the injection, the column was washed with buffer A and catalase activity was eluted from the column using a linear gradient 0–100% of buffer B (10 mM Tris-HCl pH 6.9). Aliquots containing catalase activity were then mixed and concentrated by filtration using Amicon Ultra-4, 10,000 MWCO centrifugal tubes. Aliquots of 100 μL were loaded onto a Superdex 200 10/300 GL column (GE Healthcare Life Sciences, Barcelona, Spain) and eluted using buffer C (NaCl 150 mM in 50 mM sodium phosphate buffer pH 7.0) at a flow rate of 0.5 mL.min^-1^. The separation was followed at 280 nm and column fractions were assayed routinely for catalase activity in the standard reaction medium described below.

#### Molecular mass determination

Molecular mass of the native purified enzyme was estimated using Superdex 200 10/300 GL gel filtration column, at the above mentioned conditions. Dextran blue, apoferritin (443 kDa), β- amylase (200 kDa), alcohol dehydrogenase (150 kDa), albumin (66 kDa), carbonic anhydrase (29kDa) and cobalt chloride were used as standards. The molecular mass of the monomer was determined by SDS-PAGE in the presence of 2-mercaptoethanol on a 10% acrylamide gel according to the method of Laemmli [22]. The gel was stained with Coomasie Blue and the band corresponding to the purified protein was further analyzed by mass spectrometry. SDS-PAGE image was analyzed using ImageJ software (https://imagej.net) and molecular weights were calculated using a standard curve (S1 and S2 Method).

#### Identification of the purified protein by mass spectrometry

The band corresponding to the purified protein was excised from the gel and distained with 50% acetonitrile-25 mM ammonium bicarbonate buffer (AMBIC) for 30 min at 37°C. Then, it was dried with a vacuum evaporator, reduced with 20 mM dithiothreitol (DTT) (20 min at 56°C) and alkylated with iodoacetamide 100 mM for 30 min at room temperature in the dark. The supernatant was removed and the bands were washed at 37°C, with 25 mM AMBIC pH 8.5 and then with the same buffer in 50% acetonitrile, 15 min each time. After drying, the band was incubated with 0.5 μg of Trypsin Gold Proteomics Grade (Promega Corporation, Madison, MI, U.S.A) and 0.01% ProteaseMax surfactant for 10 min at 4°C and then at 37°C for 3h. The supernatant was collected and residual peptides were washed out of the band with 50% acetonitrile in 0.5% trifluoroacetic acid (TFA) and 100% acetonitrile. Both extracts were combined, dried in a vacuum evaporator and resuspended in water/acetonitrile/formic acid (94.9:5:0.1). Analysis of the tryptic digests of the sample was carried out in an HPLC/MS system consisting of an HPLC connected to an Ion Trap XCT Plus Mass Spectrometer (Agilent Technologies, Santa Clara, CA, USA) using an electrospray interface, operating in the positive mode. Sample was injected onto a Waters XBridge BEH C18 HPLC column (Waters Corporation, USA), thermostatted at 40°C, at a flow rate of 10 μl min^-1^. After washing with water/acetonitrile/formic acid (94.9:5:0.1) the digested peptides were eluted using a linear gradient 0–80% of water/acetonitrile/formic acid (10:89.9:0.1), for 150 min. Data processing was performed with Data Analysis program for LC/MSD Trap Version 3.3 (Bruker Daltonik, GmbH, Germany) and Spectrum Mill MS Proteomics Workbench (Revision A.03.02.060B, Agilent Technologies, Santa Clara, CA, USA) and peptides were matched against *Terfezia claveryi* database from the Joint Genome Institue (https://genome.jgi.doe.gov/programs/fungi/index.jsf).

#### Sequence analysis

Sequences of several fungal catalases were collected from the following public databases: MycoCosm, GenBank, Protein Data Bank and UniprotKB. A total of 29 selected fungal sequences (S1 Table) were aligned using Clustal Omega [23]; the multiple sequence alignment obtained was cut, saving only the conserved core region (from residue 66 to 557, numbering for TcCAT-1). An unrooted phylogenetic tree was generated with MEGA 6.0 [24] using the Maximum-Likelihood method.

TcCAT-1 homology modeling was carried out with Swiss-Model [25] using as template the coordinates of a catalase from *Scytalidium thermophilum* (PDB: 4AUE). Model and template were superimposed using the command MatchMaker from UCSF Chimera package [26]. The sequences of the modeled TcCAT-1, together with those of 4AUE and catalases from *Penicillium vitale* (2XF2) and *N*. *crassa* (3EJS) and the two other catalases from *T*. *claveryi* genome ID 1091969 and ID 1248402, were aligned using Clustal Omega [23]. The alignment, was displayed using ESPript [27].

#### Biochemical characterization

Unless otherwise stated, the standard reaction medium to measure TcCAT-1 activity consisted of 1.1 U of enzyme, 10 mM H_2_O_2_ and 0.1 M sodium phosphate buffer pH 7.0, in a final volume of 1 mL. One unit of enzyme activity (U) is defined as the amount of enzyme that decomposes one μmol of H_2_O_2_ per minute at room temperature in the standard reaction medium.The reaction was started by adding the enzyme, and the decrease in absorbance at 240 nm was followed in a Jasco V-650 spectrophotometer. A blank without enzyme was prepared for each measurement. The H_2_O_2_ concentration was calculated using ε_240_ = 39.4 M^-1^cm^-1^ [28].

To determine the effect of pH, TcCAT-1 activity was followed at 25°C in the standard reaction medium using the following buffers 0.1 M: acetate (pH 4–5), phosphate (pH 5–12), Tris-HCl (pH 7–10) and carbonate (pH 10–11).

The optimum temperature was determined using the standard reaction medium described above. The measurements were recorded on a Shimadzu UV-2401PC spectrophotometer equipped with a Peltier thermoelectric temperature controlling unit (TCC-240A). Once the medium was taken to the desired temperature the reaction was started by adding the enzyme.

To study the thermostability of TcCAT-1, the enzyme was incubated at different temperatures, in phosphate buffer 0.1 M pH 7.0 for 1 h and the residual activity was measured at 25°C, in the standard reaction medium. The range of temperatures used in both assays was between 20°C and 70°C.

The effect of H_2_O_2_ concentration on TcCAT-1 activity was determined measuring the initial rate of the H_2_O_2_ consumption spectrophotometrically in the standard reaction medium changing the H_2_O_2_ concentration from 0.15 mM to 70 mM. The apparent K_m_ and V_max_ were calculated by nonlinear regression fitting of the experimental points to the Michaelis-Menten equation.

Monofunctional heme-catalases are sensitive to a number of compounds, such as hydroxylamine, 2-mercaptoethanol and 3-aminotriazole (3-AT) [18]. The inhibition of TcCAT-1 was measured using different concentrations of hydroxylamine (up to 0.5 μM), mercaptoehanol (up to 5 mM) or 3-aminotriazol (3-AT) (up to 20 mM) in the standard reaction medium. Inactivation by 3-AT was studied by incubating the enzyme with 3-AT in 0.1 M phosphate buffer pH 7.0 for one hour. At 10 min intervals, aliquots were withdrawn from this incubation media (1.1 U of enzyme per mL of 10 mM 3-AT) and the reaction was started by adding H_2_O_2_ (10 mM final concentration).

In order to evaluate if the purified TcCAT-1 presented also peroxidase activity, a reaction medium consisting of 1.1 U enzyme, 2,2'-azino-bis (3-ethylbenzothiazoline-6-sulphonic acid) (ABTS) 2 mM, H_2_O_2_ 1 mM and 0.1 acetate buffer pH 4.0, final volume 1 mL was prepared and changes in absorbance were measured at 405 nm in a Jasco V-650 spectrophotometer. Different amounts of enzyme were tested and a blank without enzyme was prepared for each measurement.

Effect of pH, temperature, substrate and inhibitor concentration and peroxidase activity assays were performed in triplicate and the mean and standard deviation were plotted.

### *TcCAT-1* expression and drought stress assay

#### Biological material

Mature ascocarps of *T*. *claveryi* were collected in Zarzadilla de Totana (Lorca, Murcia, Spain) under *H*. *almeriense* shrubs and rinsed carefully with water to remove any soil debris. Gleba and peridium were carefully separated flash frozen in liquid N_2_ and stored at -80°C until use.

Mature ascocarps collected in the above location were used to isolate *T*. *claveryi* mycelium, strain T7, which was grown *in vitro* in Erlenmeyer flasks containing Modified Melin-Norkrans optimal (MMNo) medium [29]. The cultures were shaken at 100 rpm and maintained in the dark at 23°C for three months. Then, and then mycelium was collected, flash frozen in liquid nitrogen and stored at -80°C until used.

*H*. *almeriense* seeds were collected in the same location and germinated according to Navarro-Ródenas et al. [12], with some modifications. Briefly, they were scarified and surface sterilized with 10% H_2_O_2_ (20 min) and germinated in 75 cc pots with a substrate consisting of peat moss:perlite mix, 1:1 (v:v). After two months of growth, they were transferred to 230 cc pots. Approximately 10^5^ *T*. *claveryi* spores were mixed with the new substrate and added to the pots during the transference of the plants. These spores were obtained from mature ascocarps as explained in Morte et al. [3]. Mycorrhizal status was checked by microscopy methods three months after spore inoculation and then half of the plants were subjected to drought stress before taking measurements. Non-mycorrhizal plants, both well-watered (WWNMP) and drought-stressed (DSNMP) controls were also grown. A total of six plants per treatment: well-watered mycorrhizal plants (WWMP), drought-stressed mycorrhizal plants (DSMP), WWNMP and DSNMP were obtained. Shoot water potential (Ψ) was measured periodically in order to check the water status of the plant. To this aim, 5 cm-long plant apices were covered in dark during one hour, cut and immediately placed in a pressure chamber (Soil Moisture Equipment Co; Santa Barbara, CA, U.S.A.) according to Scholander et al. [30]. Furthermore, relative soil moist content was calculated periodically by the gravimetric method [31]. It was over 75% in well watered treatments while it reached 40% in drought treatments. The moisture content was maintained at these values for all the treatments until WWMP and WWNMP showed a Ψ > -1 MPa and DSMP and DSNMP Ψ < -2 MPa, values considered as moderate drought stress for this species according to previous works [11, 12]. When reached, secondary and tertiary roots containing apical tips, were rinsed to remove soil, harvested and immediately stained and observed under an Olympus BH2 microscope, as explained later or flash frozen in liquid nitrogen and stored at -80°C for RNA extraction.

#### RNA isolation and quantitative real-time PCR

In order to study TcCAT-1 in different phases of *T*. *claveryi* life cycle and during drought stress, RNA isolation and quantitative real-time PCR was performed in several biological samples and conditions, explained before. 100 mg of WWMP and DSMP roots, mycelium and ascocarp were homogenized in liquid nitrogen with the help of mortar and pestle. RNA was extracted from secondary and tertiary roots (including apical tips) of WWMP and DSMP according to Chang et al. [32], and from mycelium cultures and ascocarps using RNAeasy Plant Mini Kit (Qiagen, Hilden, Germany Qiagen). cDNA was synthetised by Reverse-Transcrption Polymerase Chain Reaction (RT-PCR) from 0.5 μg of total RNA from each sample using QuantiTect Reverse Transcription Kit (Qiagen, Hilden, Germany), following manufacturer´s instructions.

Expression of *TcCAT-1* was studied by real-time PCR using a QuantStudioTM 5 Flex (Applied Biosystems, Foster City, California, USA). *TcCAT-1* forward (and *TcCAT-1* reverse primers were designed in the 3’untraslated region using http://www.idtdna.com (S2 Table). Each 15 μl reaction contained 1.5 μl of 1:10 cDNA template, 0.11 μl of primer mix 5 μM each and 7.5 μl of SyBR Green Master Mix (Applied biosystems, Foster City, California, USA). The PCR program consisted of 10-min incubation at 95°C, followed by 40 cycles of 15s at 95°C and 1 min at 60°C, where the fluorescence signal was measured.

The efficiency of the primer set was evaluated by performing real-time PCR on several dilutions of cDNA. Real-time PCR threshold cycle (Ct) was determined in triplicate. 2^-ΔΔCt^ method was used to evaluate the expression of each gene [33] normalizing gene expression to the geometric mean of elongation factor (EF1-alfaII) (JF491354, NCBI) and actin (ID1089750, Mycocosm) levels (S2 Table). These genes have been confirmed as a proper reference genes in different conditions using transcriptomics analyses (unpublished results) and geNorm, included in qbase+ software, version 3.0 (Biogazelle, Zwijnaarde, Belgium (www.qbaseplus.com). All primers were tested on all different samples to test specificity and cross amplification (S2 Table). Real-time PCR experiments were carried out in six separate biological samples and non-template controls were performed in all PCR reactions.

#### Hydrogen peroxide content

150 mg of roots from WWMP, WWNMP, DSMP and DSNMP treatments were homogenized in liquid nitrogen using mortar and pestle. They were resuspended in 1 mL of phosphate 0.1 M buffer containing 0.1% trichloroacetic acid (TCA). They were centrifuged at 12000 *g* for 10 min and pellet was discarded. Supernatant was diluted and H_2_O_2_ content was measured using a fluorimetric H_2_O_2_ assay kit (Sigma, Madrid, Spain). The protein content was measured according to the bicinchoninic acid method [34] using BSA as standard. H_2_O_2_ content was normalized using total protein of the extract. Six biological replicates of each treatment were measured.

#### Fungal colonization

Fungal colonization of each plant (n = 6) for every treatment: WWMP, DSMP, WWNMP and DSNMP was measured under an Olympus BH2 microscope, after staining their roots with trypan blue as described in Gutiérrez et al. [35]. To calculate the mycorrhization status, 100 secondary and tertiary root sections per sample were observed under the microscope and were classified as mycorrhizal or nonmycorrhizal depending on the presence/absence of *T*. *claveryi* mycorrizal structures.

#### Statistical analyses

*TcCAT-1* expression, H_2_O_2_ content and fungal colonization were subjected to analysis of the variance (ANOVA) and to Tukey test using R studio (version 1.1.456). Before the analysis, data were subjected to normality (Shaphiro-Wilk) and homoscedasticity (Levene) tests. Data plotting was carried out using Sigmaplot v 10.0 (Systat Software, UK).

## Results

### Biochemical and sequence analysis

#### Enzyme extraction and purification

Ascocarp crude extract TX-114 phase partitioning resulted in a two-fold purification (Table 1). Submitting the aqueous phase to ultrafiltration removed 63% of the proteins. Loading the enzyme onto HiTrap Phenyl HP 1 mL column resulted in a 23.2 fold purification and a recovery of 71.2% of total activity, while TcCAT-1 was finally purified to apparent homogeneity using gel filtration chromatography. By this final step, 33.7-fold purification was obtained and the residual activity was 19.3%. The purified catalase was stable at -20°C for at least one month.

**Table 1:** Purification of *T*. *claveryi* CAT (2 g of gleba).

Purification step	Volume (mL)	Total activity (U)	Total protein (mg)	Specific activity (U/ mg)	Recovery (%)	Purification (fold)
**Crude extract**	8.0	303.8	34.7	8.8	100	1
**10%TX-114 supernatant**	4.5	296.8	16.7	17.8	97.7	2.0
**100K MWCO filtration**	1.0	267.4	6.2	43.1	88.0	4.9
**HiTrap Phenyl HP**	4.5	216.3	1.1	202.7	71.2	23.2
**Superdex 200 10/300 GL**	2.0	58.8	0.2	295.4	19.3	33.7

#### Molecular mass determination

**Fig 1:**
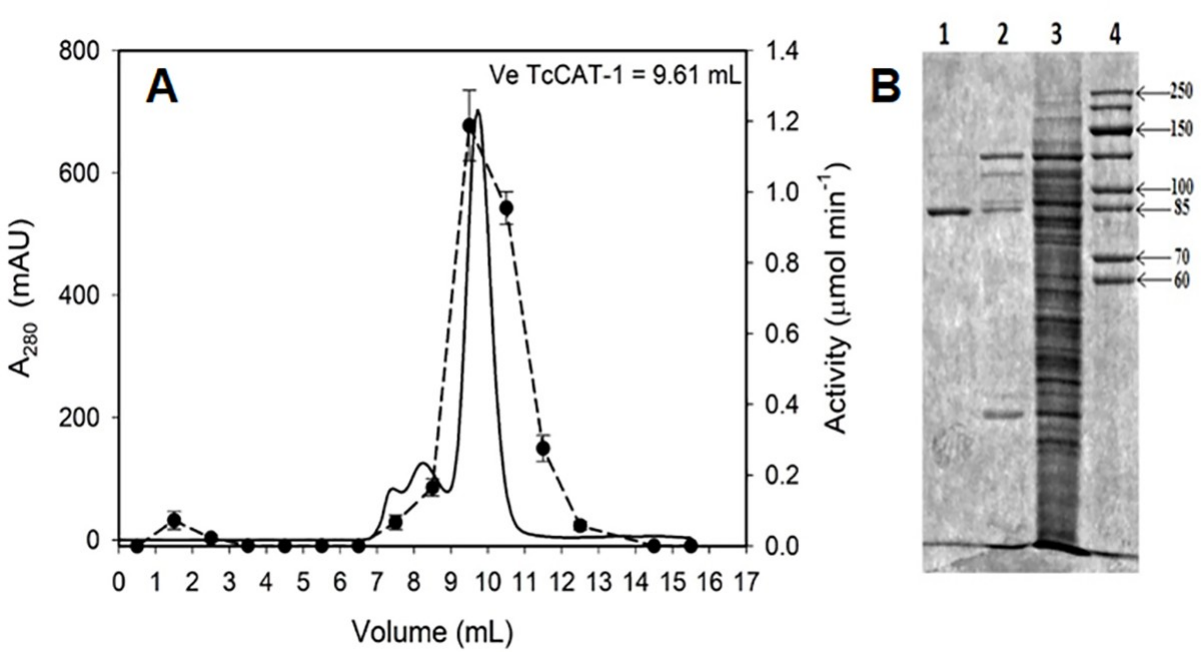
Molecular mass determination of TcCAT-1. (A) Gel filtration chromatogram. Absorbance at 280 nm is represented with solid line and TcCAT-1 activity measured in the standard reaction medium is represented with dashed line. (B) SDS-PAGE (10%). Lane 1: CAT eluted in Superdex 200 10/30 GL. Lane 2: CAT eluted in HiTrap Phenyl HP. Lane 3: Crude extract. Lane 4: SDS unstained molecular mass marker, mass indicated in kilodaltons.

The MW of the purified catalase was estimated to be 357.8 kDa by gel filtration (Fig 1A). When the enzyme was analyzed by SDS-PAGE, a value of 90.4 kDa for the monomer was obtained (Fig 1B).

#### pH and temperature

**Fig 2:**
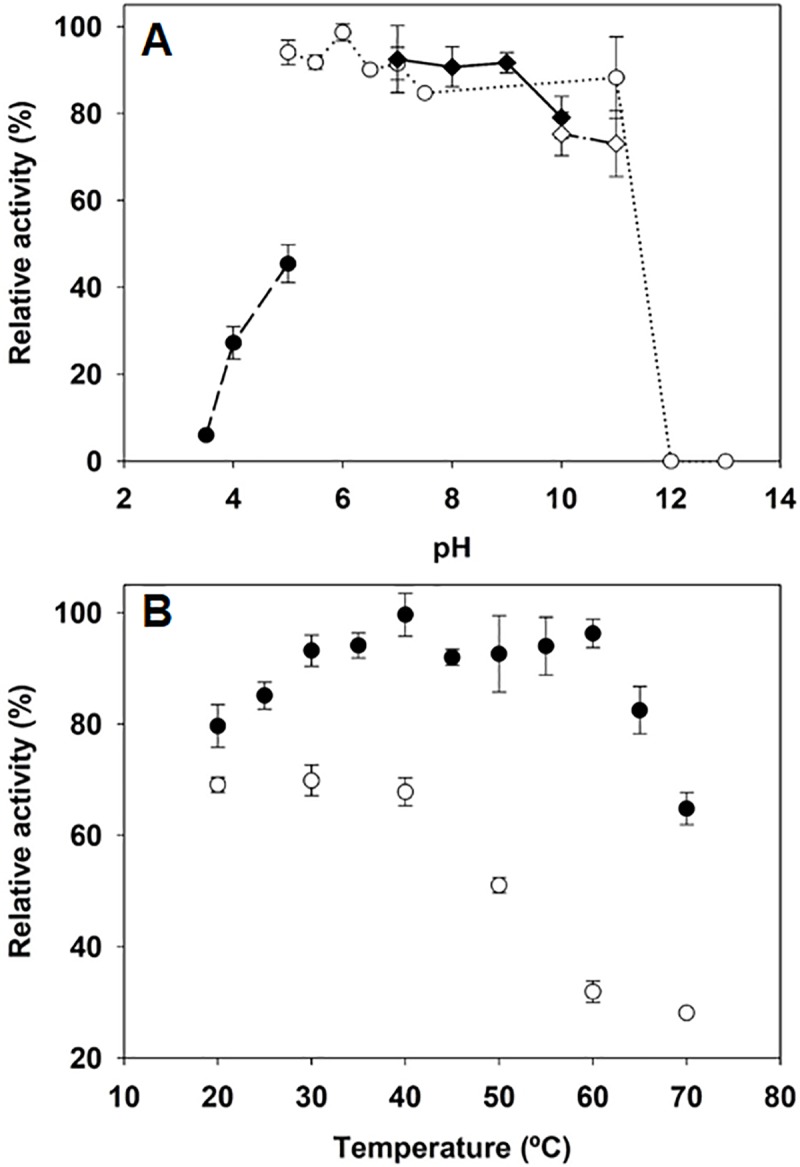
**Effect of pH (A) and temperature (B) on CAT activity.** (A) Acetate (●), phosphate (○), Tris-HCl (♦) and carbonate (◊) buffers are represented. (B) Determination of activity at optimum temperature (●) and activity after 60 min of incubation at each temperature (○). Values are the mean of 3 replicates. Bars indicate standard error.

TcCAT-1 remained maximally active over a broad range of pH (from 5–11) using phosphate buffer and no optimal pH was observed. No activity was detected at pH 12.0 (Fig 2A). The enzyme showed very little activity at acid pHs. This decrease in activity is also caused by acetate buffer, since the activity at pH 5.0 was considerably higher when acetate was replaced by phosphate. The effect of temperature on the initial rate of TcCAT-1 (Fig 2B) indicates that, in a range from 20°C to 65°C, TcCAT-1 remains maximally active and presents a relative activity between 80–100%, losing only 32% of activity at 70°C. Thermostability tests showed a loss of 50% of enzyme activity (T_50_) around 50°C and that the enzyme retains 28% of activity after one hour at 70°C (Fig 2B).

#### Substrate concentration

**Fig 3:**
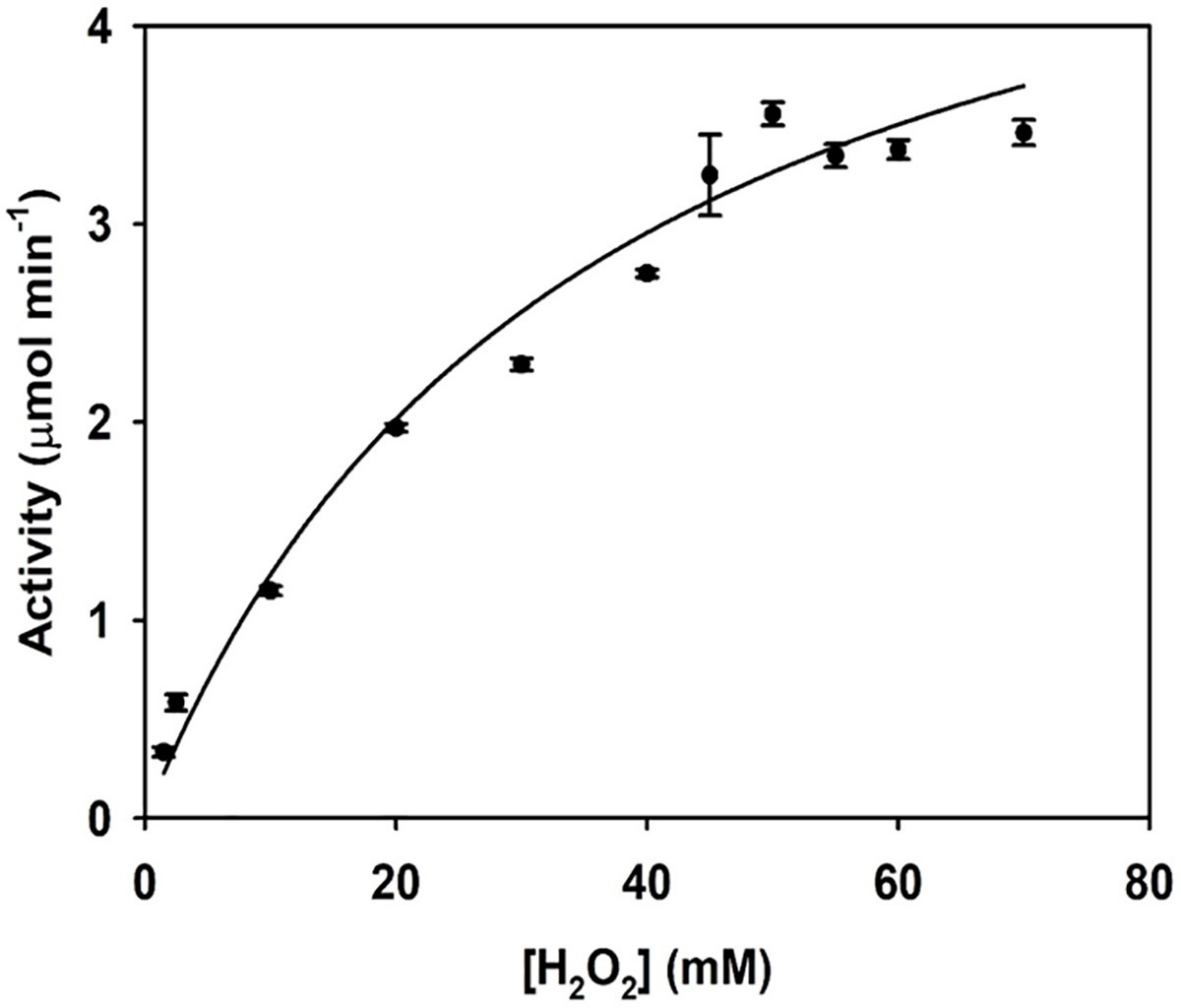
Effect of substrate concentration on TcCAT-1 activity. Values are the mean of 3 replicates. Bars indicate standard error.

The effect of H_2_O_2_ concentration on the initial rate of TcCAT-1 was analyzed spectrophotometrically increasing the substrate concentration up to 70 mM. The experimental results were fitted to the Michaelis-Menten equation and a good correlation was observed (Fig 3). The values obtained for the apparent K_m_ and V_max_ were 35.5 mM and 5.5 μmol min^-1^, respectively.

#### Inhibitors

The concentrations of inhibitor required for 50% inhibition (IC_50_) calculated for hydroxylamine was 0.1 μM and 0.125 mM for 2-mercaptoethanol (Table 2). The inhibition produced in TcCAT-1 activity by the presence of 3-AT (up to 20 mM) in the reaction medium was only of 10%. However, a time-dependent decrease in the activity of TcCAT-1 was observed when the enzyme was incubated in the presence of 10 mM 3-AT (Table 2). In these conditions, 50% of activity was lost in 10 min. Peroxidase-like activity was not detected using ABTS and H_2_O_2_ as substrates.

**Table 2:** Effect of inhibitor on TcCAT-1 activity from *T*. *claveryi*.

Inhibitor	IC_50_
Hydroxilamine	0.10 ± 0.01μM
2-mercaptoethanol	0.13 ± 0.02 mM
3-Aminotriazol	10 mM, after 10 min incubation

See Material and methods for details.

#### Sequence identification

The genome of *T*. *claveryi* has been recently published by Joint Genome Institute (JGI, California, USA) (https://genome.jgi.doe.gov/programs/fungi/index.jsf). Three different sequences coding for catalases were found in this genome (proteins ID 1216276, 1091969 and 1248402). The Protein Mass Fingerprinting (PMF) presented a 41% sequence coverage and 25 matched peptides with protein ID 1216276 automatically annotated as a “mono-functional catalase” in that database and did not match with any of the other two annotated catalases from *T*. *claveryi* genome (ID 1091969 and ID 1248402).

The percentage of sequence identity of TcCAT-1 with the other two *T*. *claveryi* catalases, determined by multiple sequence alignment, was 44.7% for ID 1248402 (76.7% of coverage) and 24.9% for ID 1091969 (52.3% coverage).

**Fig 4:**
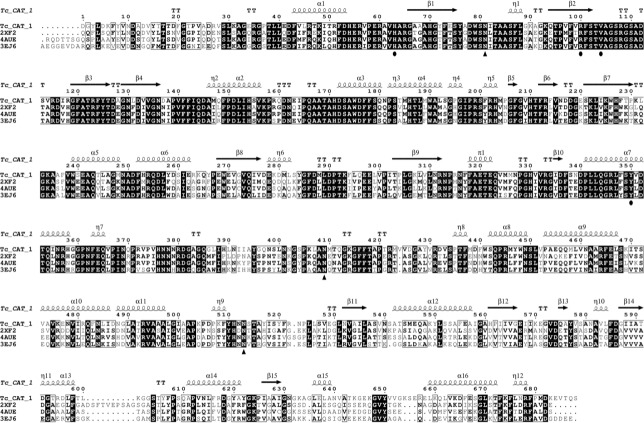
Multiple sequence alignment of TcCAT-1 with other fungal catalases. *Scytalidium thermophilum* (4AUE), *Neurospora crassa* Cat-3 (3EJ6) and *Penicillium vitale* (2XF2). The alignment was carried out with Clustal Omega [23] and the output was generated with ESPript 3.9 [27]. The secondary structure corresponding to the modeled TcCAT-1 is displayed above, where springs represent α helix or 3_10_ helix (η) and arrows β strands. White characters on a black backround indicate residues strictly conserved. (●) represents the conserved key residues, while (▲) represents the glycosilation sites, predicted in TcCAT-1 and observed in 3EJ6.

**Fig 5:**
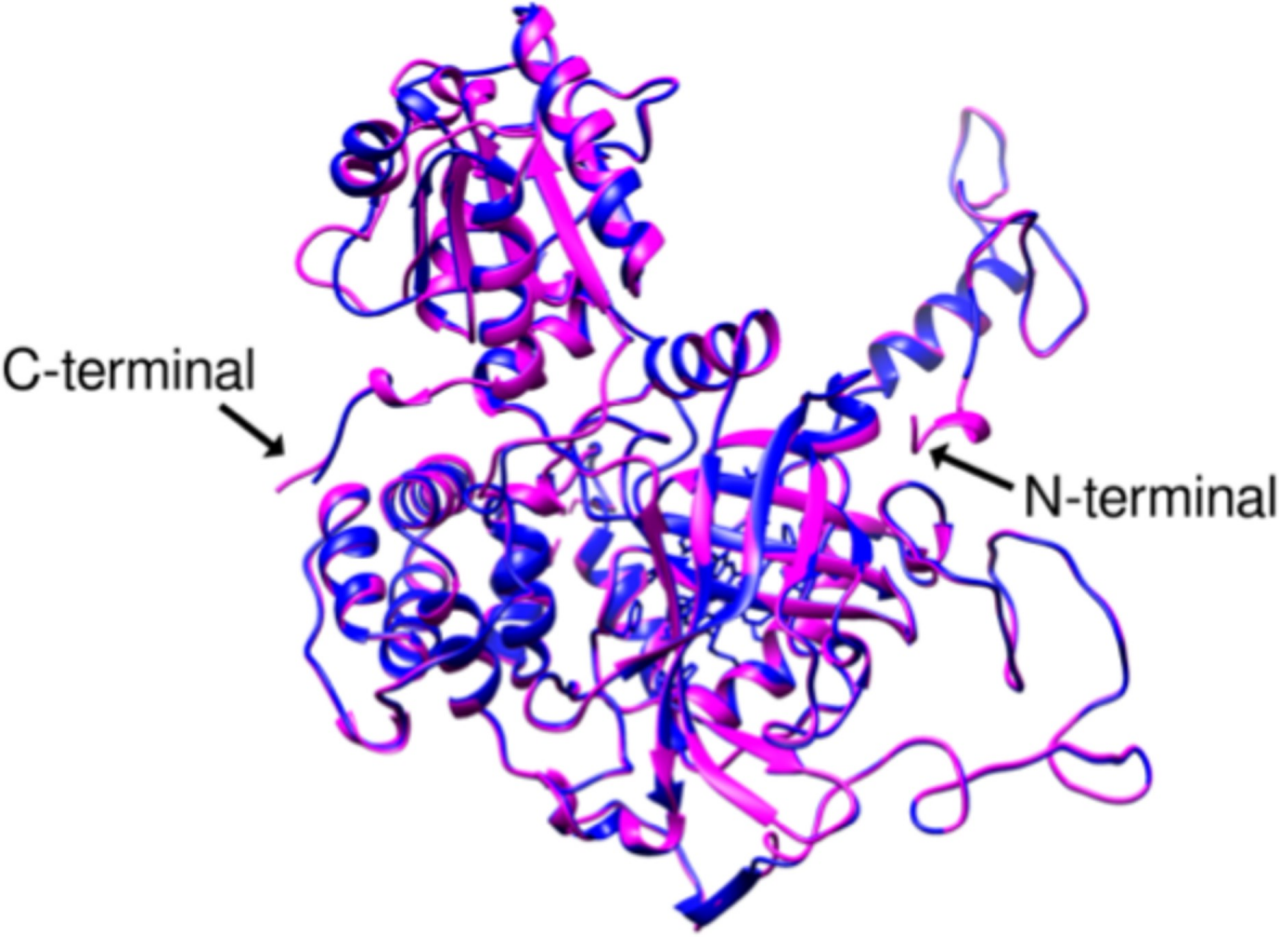
Main-chain superposition of TcCat-1 model (magenta) and 4AUE (blue).

The multiple sequence alignment of TcCAT-1 (ID 1216276) and fungal catalases from other species (Fig 4) shows a high percentage of identity (62% with *Scytalidum thermophilum* (4AUE) and 58% with *Penicillium vitale* (2XF2) and *Neurospora crassa* (3EJ6)). This high percentage of sequence identity with other catalases of known structure allowed to build a homology model of TcCAT-1, the topology diagram of which is shown on top of Fig 4. Fig 5 shows main-chain superposition of TcCAT-1 model and its template (4AUE). The overall structure of the modeled monomer is similar to those described for other catalases [36].

#### Phylogenetic analysis

**Fig 6:**
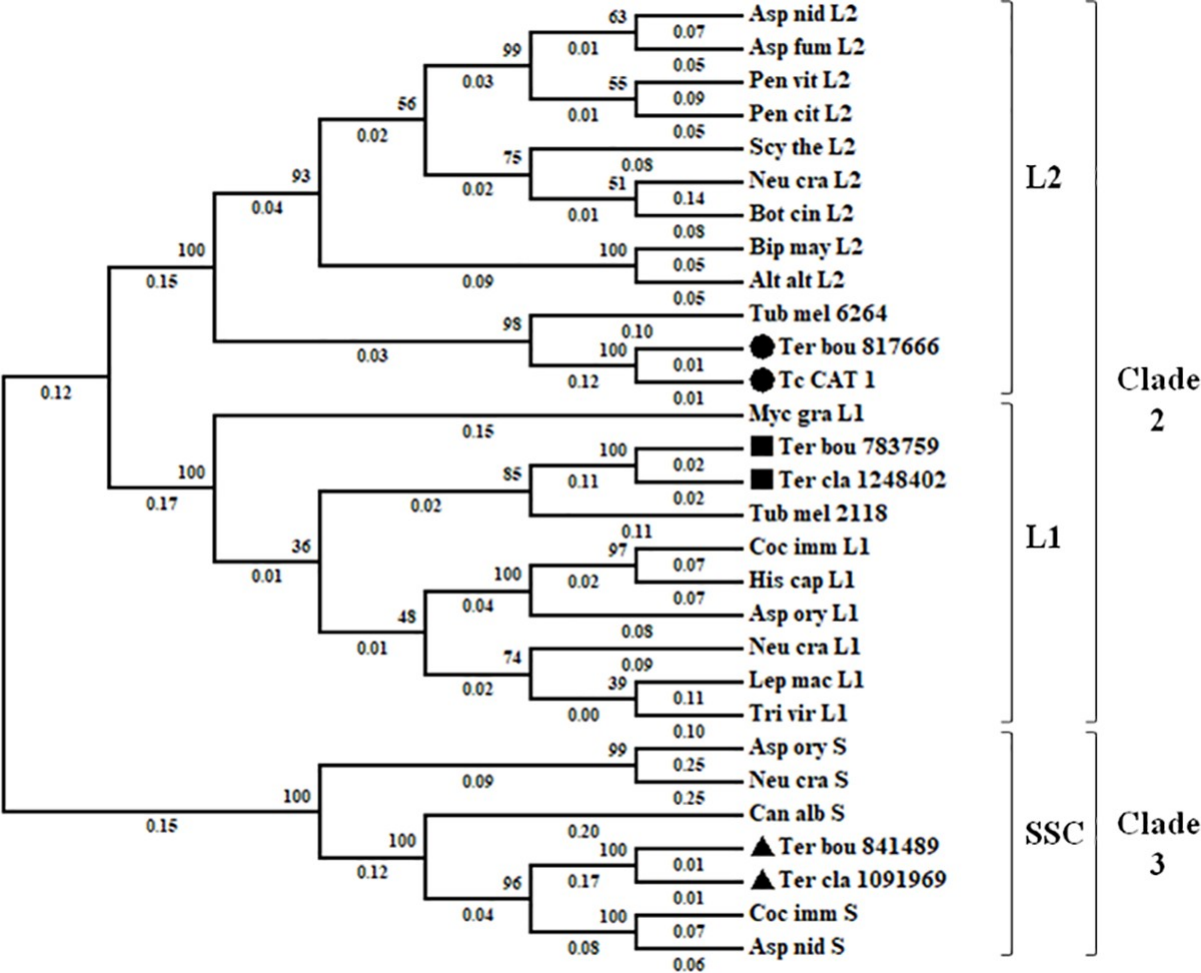
Maximum-Likelihood unrooted tree of fungal catalases. *Terfezia claveryi* and *Terfezia boudieri* novel catalases were identified as L1 (■), L2 (●) or SSC (▲).The percentage of replicate trees in which the associated taxa clustered together in the bootstrap test (1000 replicates) and the branch length are shown next to and below the branches, respectively.

In order to gain more information on TcCAT-1, a phylogenetic tree was built using sequences of catalases from truffles (*T*. *claveryi*, *Terfezia boudieri* and *Tuber melanosporum*) and other Ascomyota (Fig 6). TcCAT-1 has been identified as a catalase belonging to L2 group.

### *TcCAT-1* expression and drought stress assay

#### *TcCAT-1* mRNA levels during *T*. *claveryi* life cycle

**Fig 7:**
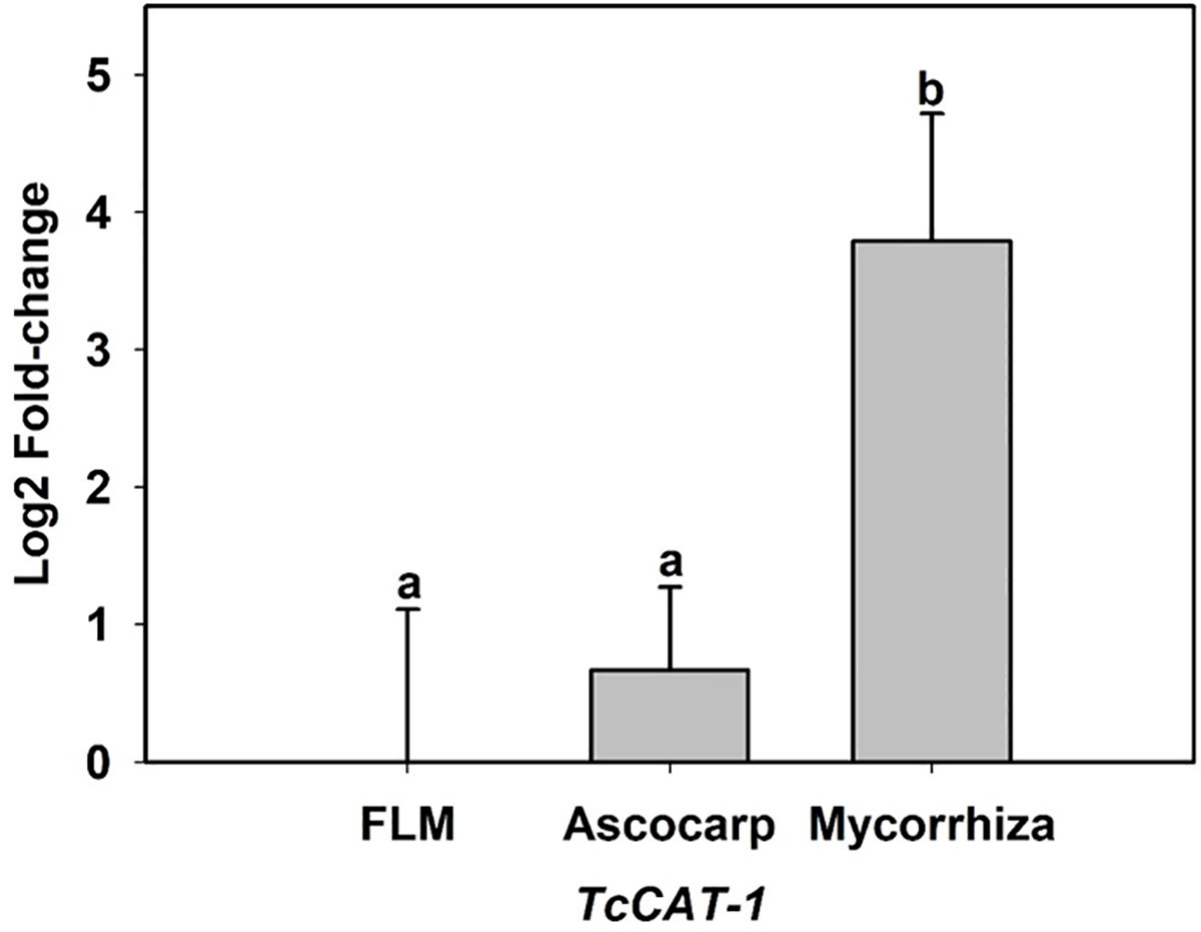
Log2 fold-change of *TcCAT-1* during different stages of *T*. *claveryi* biological cycle. FLM, free living mycelium; WWMP, well-watered mycorrhizal plants. Fold-change data was calculated by normalizing to actin (ID1089750, Mycocosm) and elongation factor (JF491354, NCBI) levels. Bars show the means ± standard error (*n* = 6). Different letters indicate different significance groups when *p* < 0.05, as determined by analysis of variance (ANOVA) and a Tukey test.

Analysis of RT-qPCR revealed the lowest expression levels in free living mycelium (FLM) and no significant fold-change in the ascocarp. mRNA levels of *TcCAT-1* were up-regulated in WWMP showing a 3.79 Log2 fold-change (Fig 7). No amplification was obtained when using *TcCAT-1* primers in non-mycorrhizal plants (S2 Table).

#### *TcCAT-1* mRNA levels and H_2_O_2_ content during drought stress

**Fig 8:**
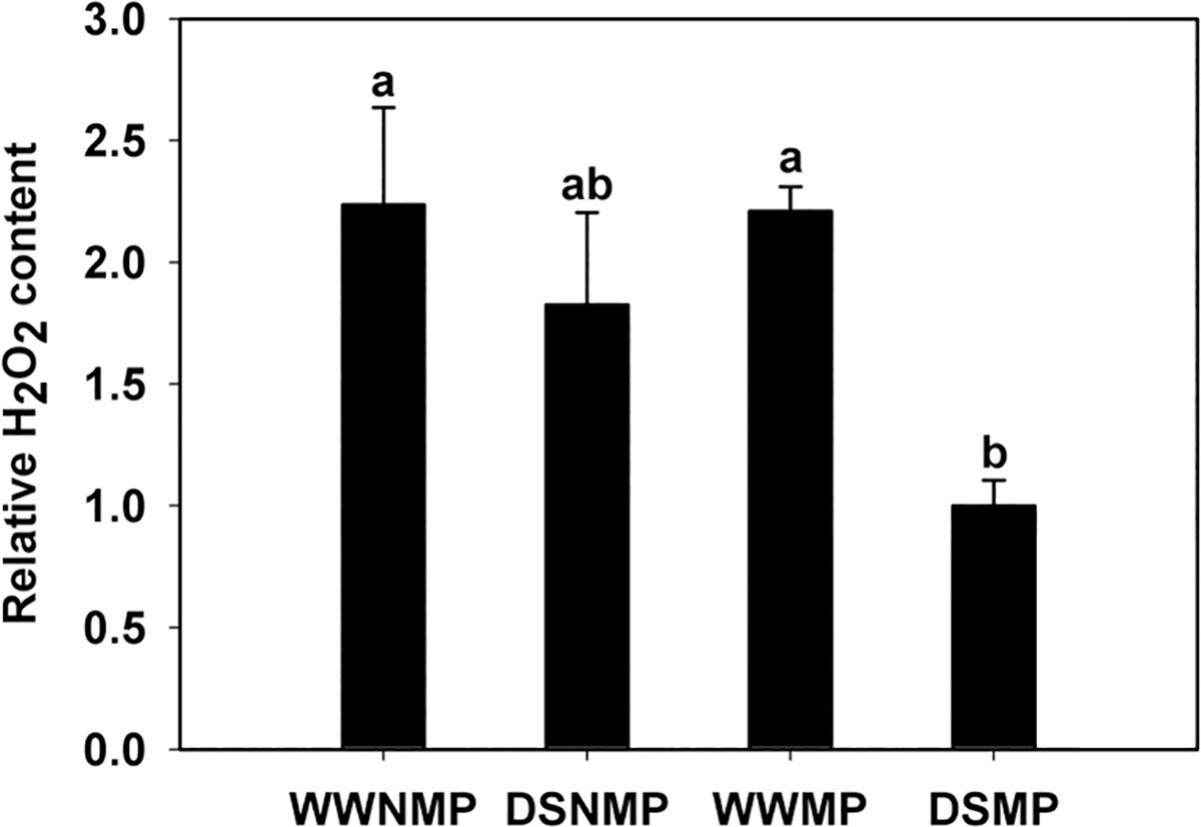
Relative H_2_O_2_ content in roots of plants with different mycorrhiza and water treatments. Bars show the means ± standard error (*n* = 6). Different letters indicate different significance groups between treatments when p < 0.05, as determined by analysis of variance (ANOVA) and a Tukey test. Mycorrhizal treatments: WWMP, well-watered mycorrhizal plants; DSMP, drought-stressed mycorrhizal plants. Non-mycorrhizal treatments: WWNMP, well-watered non-mycorrhizal plant; DSNMP, drought-stressed non-mycorrhizal plant.

When comparing *TcCAT-1* expression between WWMP and DSMP, no significant change was observed (S3 Table). At the same time, H_2_O_2_ content in roots was significantly lower only in DSMP compared to well-watered treatments (Fig 8).

#### Fungal colonization during drought stress

**Fig 9:**
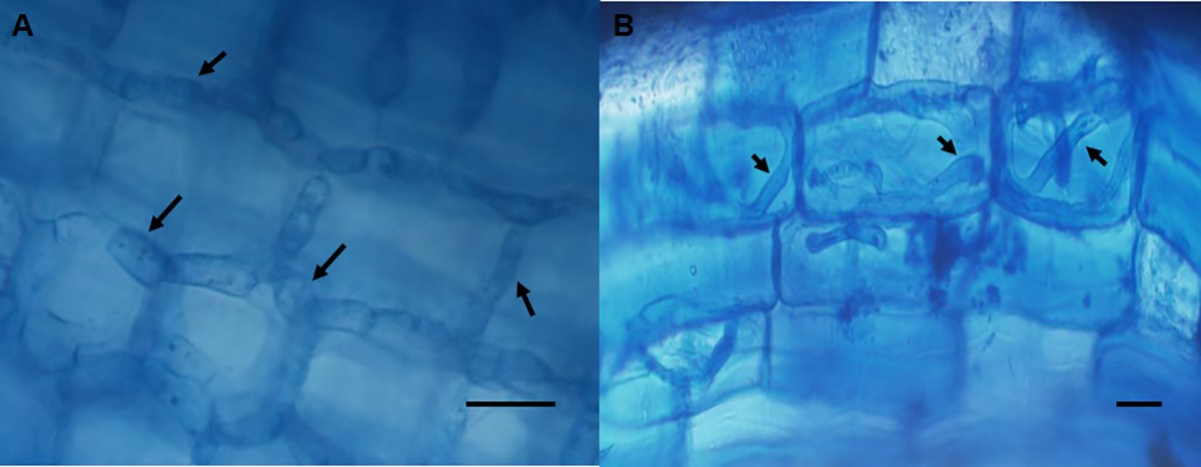
Fungal colonization of *T*. *claveryi* in *H*. *almeriense* roots. Arrows indicate inter- or intracellular hyphae and scale bar represents 20 μm. (A) Intercellular colonization. (B) Intracellular colonization.

Fungal colonization in each treatment was measured, finding a higher mycorrhization percentage in DSMP (48.8%) than in WWMP (27.8%), while no colonization was observed in non-mycorrhizal controls (Table 3). Along the root system, ecto- (Fig 9A), ectendo- and endo- (Fig 9B) mycorrhizal structures were observed.

**Table 3:** Fungal colonization of *H*. *almeriense* x *T*. *claveryi* mycorrhizal plants.

Sample	Mycorrhization percentage (%) ± Standard Error
**WWMP**	27.8 ± 5.8 a
**DSMP**	48.8 ± 6.4 b
**WWNMP**	Mycorrhiza undetected
**DSNMP**	Mycorrhiza undetected

WWMP, well-watered mycorrhizal plants; DSMP, drought-stressed mycorrhizal plants; WWNMP, well-watered non-mycorrhizal plants; DSNMP, drought-stressed non-mycorrhizal plants. Different letters indicate different significance groups when *p* < 0.05, as determined by Student’s t-test.

## Discussion

We have purified TcCAT-1 from ascocarps of *T*. *claveryi* and performed a biochemical and sequence analysis. The presence of phenolic compounds and other metabolites, which can bind to proteins and modify their properties, difficult enzyme purification in fungal extracts. In addition, *T*. *claveryi* ascocarps are rich in lipids which impair spectrophotometric measurements in crude extracts. To remove all these compounds, phase partitioning with TX-114 was used since this method gave good results with other enzymes from this fungus [37–40]. The whole process of purification (Table 1) was considered good according to the yield and purification levels and when compared to other catalase purifications [41, 42].

Molecular weights obtained by gel chromatography and by SDS-PAGE (Fig 1) suggest that TcCAT-1 is a homotetramer and belongs to the LSCs group of monofunctional heme-catalases. Gel filtration separates molecules according to size, which is dependent on both molecular weight and shape. Thus, the shape of the protein could affect the molecular weight measured by gel filtration and may explain, in part, the discrepancies found between the molecular weights of the monomer and tetramer determined experimentally. On the other hand, the experimental MW of this tetramer is very similar to the 354 kDa determined for Cat-1 from *Neurospora crassa* [13]. The MW of this sequence was estimated as 82,017 Da with the ProtParam tool from Expasy [43]. The differences between the estimated and the experimental MW (90.4kDa) are very similar to the described above for the glycoprotein Cat-1 from *N*. *crassa* [13], thus suggesting that TcCAT-1 is also a glycoprotein. In fact, when TcCAT-1 sequence was analyzed with ScanProsite tool [44] three predicted sites of N-glycosilation were detected. The alignment (Fig 4) confirms the conservation in the sequence of TcCAT-1 of the N-glycosilation sites reported for *N*. *crassa* Cat-3 [36]. Conserved residues marked in Fig 4 are characteristic of the catalase family. For example, catalytic histidine H62, stabilized by an arginine (R100) and a valine (V104) and a tyrosine (Y350), which serves as the heme proximal side ligand, are within conserved regions.

TcCAT-1 belonged to L2 group (Fig 6) when a phylogenic analysis was performed. With exceptions, such as the plant pathogen *Ustilago maydis* that only has a catalase-peroxidase but no catalase genes [45], most Ascomycota have several monofunctional heme catalases, usually one to four SSCs and two LSCs, one L1 and one L2. In addition to TcCAT1, other two catalases, ID 1248402 and ID 1091969, with low sequence identity to TcCAT1 (no higher than 24.9%) have been found in *T*. *claveryi* fungal genome, the former belonging to L1 and the latter to the SSC phylogenetic groups (Fig 6).

The pH, optimal temperature profile and thermostability showed by TcCAT-1 (Fig 2) is typical of monofunctional catalases [13, 46, 47]. The apparent K_m_ (Fig 3) is similar to the estimated for CAT-1 of *N*. *crassa* at concentrations of H_2_O_2_ below 100 mM [13] and to those described for CAT from the bacteria *P*. *aeruginosa* [18]. The sensitivity of TcCAT-1 to hydroxylamine (Table 2) is similar to the reported for CAT from *Aspergillus niger*, *E*. *coli or N*. *crassa* (IC_50_ of 0.4, 0.12 and 0.19 respectively) [13,18], while its sensitivity to 3-AT (Table 2) is similar to the reported by Díaz et al. [21] for CAT-1 from *N*. *crassa*.

Catalase activity in fungi has been related to stress conditions (high and low water content, high and low temperature, presence of competing species, etc) and also to development of their life cycle [19], in the case of edible mycorrhizal fungi it consists on a free-living mycelium phase, a symbiotic mycorrhizal phase and a fruiting body phase [48], but the role of the vast majority of these fungal enzymes remains to be elucidated.

The saprophytic edible fungi *Pleorotus ostreatus* and the entomopathogen *Beauveria bassiana*, present both several catalases with different expression levels, in different life cycle stages, suggesting distinct functions during their life cycle [49, 50]. However, until now the expression and function of fungal catalases during mycorrhiza life-cycle has not been studied. The upregulation of *TcCAT-1* in mature mycorrhiza reported in this paper (Fig 7) suggests that this enzyme may be playing a role in mycorrhizal symbiosis of *T*. *claveryi* and *H*. *almeriense*. In any case, mRNA data should be treated with caution, since it has been shown that, at steady-state, mRNA levels mainly explain protein concentrations, while during dynamic phases such as cellular differentiation or stress response, post-transcriptional regulation may deviate this ideal correlation between transcript and protein levels [51].

In AM associations, the accumulation of ROS and the induction of plant ROS-scavenging enzymes, such as superoxide dismutase, catalase and peroxidase during the mycorrhization process have been reported [14,52]. Baptista et al. [15] reported an increase in H_2_O_2_ production, with a pattern similar to the observed for pathogenic infections, during early stages of ECM establishment between *Castanea sativa* and *Pisolithus tinctorius*. However, in order to establish a successful association, the fungal symbiont must be able to elude the host´s defense response and this response could explain the higher expression level of *TcCAT-1* in *H*. *almeriense* x *T*. *claveryi* mycorrhizal roots. Phylogenetic analysis reinforces this idea since it shows that TcCAT1 clusters with L2 catalases, which are usually extracellular enzymes induced by different stressors [19].

Catalases are the main responsible for the dismutation of the H_2_O_2_, one of the ROS that can be produced during drought stress [8]. One of the advantages of mycorrhization is to favor the adaptation of the host plant to water deficit through various processes, being protection against oxidative damage one of them. In this sense, different studies have reported decreases in H_2_O_2_ content, usually correlated with an induction in catalase activity, in mycorrhizal roots undergoing drought stress [9, 53]. At the same time, it is already known that *T*. *claveryi* mitigates the negative effects of drought by physiological and nutritional alterations in *H*. *almeriense* [11, 12], However, to what extent drought induces oxidative stress in *H*. *almeriense* has not been investigated yet. The results here presented are the first approach of how oxidative stress is managed by the *H*. *almeriense x T*. *claveryi* mycorrhiza in water-deficit conditions. A significant decrease in the H_2_O_2_ root content is observed only in mycorrhizal plants submitted to drought stress (Fig 8). The increase in mycorrhization levels (Table 3) could play a role in the H_2_O_2_ decrease. This decrease is not observed in non-mycorrhizal plants under drought stres (Fig 8), suggesting that the beneficial effects of mycorrhiza during water deficit at cellular level may involve a relief of oxidative stress. However, there is no increase in TcCAT-1 expression levels (S3 Table). This result suggests that TcCAT-1 is not responsible for the decrease in the H_2_O_2_ levels observed in DSMP and other ROS scavenger enzymes or even antioxidant compounds may be involved. Future and more exhaustive research will be needed to fully comprehend the role that plant and fungal enzymes and/or non-enzymatic compounds may have in the oxidative response of the mycorrhiza against the drought.

In conclusion, TcCAT-1 purified and characterized in this study shows an interesting expression pattern during the life cycle of *T*. *claveryi*. It seems to play a significant role in the mycorrhizal symbiosis of *T*. *claveryi* and *H*. *almeriense* since it is upregulated in mature mycorrhiza compared to free living mycelium and fruit body stage. Mycorrhization has a beneficial effect in terms of oxidative stress relief, but the transcriptional profile of this enzyme does not seem to be the responsible of the H_2_O_2_ decrease observed in the mycorrhizal plants during water deficit. Future investigation would be, thus, needed to assess the role of TcCAT-1 and other fungal ROS scavengers in mediating mycorrhiza formation and plant resistance to drought stress.

## Supporting information

S1 Table Catalase fungal sequences used in the phylogenetic tree. (DOCX)

S2 Table Primers used and cross amplification test. (DOCX)

S3 Table TcCAT-1 expression pattern in WWMP and DSMP. (DOCX)

S1 Method Calculation of native molecular weight by gel filtration. (XLSX)

S2 Method Calculation of molecular weight of the monomer by SDS-PAGE. (XLSX)
