# Supplementary material for: Purification and characterization of Terfezia claveryi TcCAT-1, a desert truffle catalase upregulated in mycorrhizal symbiosis
Source: PLoS One. 2019 Jul 10;14(7):e0219300. doi: 10.1371/journal.pone.0219300 (PMC6620010; doi:10.1371/journal.pone.0219300)
Supplement: S1 Table — (DOCX) [file pone.0219300.s001.docx]

**S1 Table. Catalase fungal sequences used in the phylogenetic tree.**

| Species | Name | Database | Accesion Number |
| --- | --- | --- | --- |
| *Alternaria alternata* | Alt_alt_L2 | NCBI/GenBank | OWY51759.1 |
| *Aspergillus fumigatus* | Asp_fum_L2 | NCBI/RefSeq | XP_748550 |
| *Aspergillus nidulans* | Asp_nid_L2 | NCBI/RefSeq | XP_682608.1 |
| *Aspergillus nidulans* | Asp_nid_S | NCBI/GenBank | AAG45152.2 |
| *Aspergillus oryzae* | Asp_ory_L1 | NCBI/GenBank | BAC56946.1 |
| *Aspergillus oryzae* | Asp_ory_S | NCBI/GenBank | BAE63583.1 |
| *Bipolaris_maydis* | Bip_may_L2 | NCBI/RefSeq | XP_014078299.1 |
| *Botrytis cinerea* | Bot_cin_L2 | NCBI/GenBank | AAK77951.1 |
| *Candida albicans* | Can_alb_S | NCBI/RefSeq | XP_718818.1 |
| *Coccidioides immitis* | Coc_imm_L1 | UniportKB | J3K0Y0 |
| *Coccidioides immitis* | Coc_imm_S | NCBI/GenBank | EAS33312.3 |
| *Histoplasma capsulatum* | His_cap_L1 | NCBI/Genbank | AAF01462.1 |
| *Leptosphaeria maculans* | Lep_mac_L1 | NCBI/RefSeq | XP_003841429.1 |
| *Mycosphaerella graminicola* | Myc_gra_L1 | Mycocosm/JGI | 98331 |
| *Neurospora crassa* | Neu_cra_L1 | UniportKB | Q9C168 |
| *Neurospora crassa* | Neu_cra_L2 | PDB | 3EJ6 |
| *Neurospora crassa* | Neu_cra_S | NCBI/GenBank | EAA32637.1 |
| *Penicillium citrinum* | Pen_cit_L2 | NCBI/GenBank | ABB89950.1 |
| *Penicillium vitale* | Pen_vit_ L2 | PDB | 2XF2 |
| *Scytalidum thermophilum* | Scy_the_L2 | PDB | 4AUE |
| *Terfezia boudieri* | Ter_bou_783759 | Mycocosm/JGI | 783759 |
| *Terfezia boudieri* | Ter_bou_817666 | Mycocosm/JGI | 817666 |
| *Terfezia boudieri* | Ter_bou_841489 | Mycocosm/JGI | 841489 |
| *Terfezia claveryi* | Ter_cla_1091969 | Mycocosm/JGI | 1091969 |
| *Terfezia claveryi* | Tc_CAT_1 | Mycocosm/JGI | 1216276 |
| *Terfezia claveryi* | Ter_cla_1248402 | Mycocosm/JGI | 1248402 |
| *Trichoderma virens* | Tri_vir_L1 | Mycocosm/JGI | 38844 |
| *Tuber melanosporum* | Tub_mel_6264 | Mycocosm/JGI | 6264 |
| *Tuber melanosporum* | Tub_mel_2118 | Mycocosm/JGI | 2118 |
