# Supplementary material for: Purification and characterization of Terfezia claveryi TcCAT-1, a desert truffle catalase upregulated in mycorrhizal symbiosis
Source: PLoS One. 2019 Jul 10;14(7):e0219300. doi: 10.1371/journal.pone.0219300 (PMC6620010; doi:10.1371/journal.pone.0219300)
Supplement: S2 Table — (DOCX) [file pone.0219300.s002.docx]

**S2 Table. Primers used in the study, *geNorm* M score and cross amplification test.**

| Primer | Gene | Sequence (forward and reverse respectively) | Efficiency (%) * | Stability (geNorm)* | Sample | Ct mean (3 biological replicates) * |
| --- | --- | --- | --- | --- | --- | --- |
| TcCAT-1 | Catalase | 5’GGAGAGGGTTGGTGTAATTCTT’3  5’GCTGCCCTTGATAACCCTATT’3 | 80.9 | nd | NMP | Undetermined |
|  |  |  |  |  | FLM | 25.365 ± 0.177 |
|  |  |  |  |  | Ascocarp | 24.742 ± 0.707 |
|  |  |  |  |  | WWMP | 28.150 ± 0.584 |
|  |  |  |  |  | DSMP | 28.590 ± 0.671 |
| TcActin | Actin | 5’CACTGGAGCATGGGATTGT’3  5’GTACTGGATGCTCCTCAGAAAG’3 | 99.5 | 0.342 | NMP | Undetermined |
|  |  |  |  |  | FLM | 26.367 ± 0.182 |
|  |  |  |  |  | Ascocarp | 26.537 ± 0.308 |
|  |  |  |  |  | WWMP | 33.525 ± 0.880 |
|  |  |  |  |  | DSMP | 33.404 ± 0.820 |
| TcEF | Elongation Factor | 5’TCCGTTAAGGAAATTCGTCG’3  5’GTCCAGGGTGGTTCATCAAG’3 | 97.71 | 0.312 | NMP | Undetermined |
|  |  |  |  |  | FLM | 21.042 ± 0.720 |
|  |  |  |  |  | Ascocarp | 20.655 ± 0.182 |
|  |  |  |  |  | WWMP | 30.686 ± 0.915 |
|  |  |  |  |  | DSMP | 30.674 ± 0.884 |

NMP = Non-mycorrhizal plants; FLM = Free living mycelium.* An efficiency of 100% means that every cycle there are 2 times the previous cycle number of copies; Stability or M score is a value obtained using geNorm software to select the most stable reference gene/s, values under 0.5 are considered stable for reference genes; The Ct (cycle threshold) is defined as the number of cycles required for the fluorescent signal to cross the threshold.
