# Supplementary material for: Purification and characterization of Terfezia claveryi TcCAT-1, a desert truffle catalase upregulated in mycorrhizal symbiosis
Source: PLoS One. 2019 Jul 10;14(7):e0219300. doi: 10.1371/journal.pone.0219300 (PMC6620010; doi:10.1371/journal.pone.0219300)
Supplement: S3 Table — (DOCX) [file pone.0219300.s003.docx]

**S3 Table. TcCAT-1 expression pattern in WWMP and DSMP.**

| Sample | Relative expression |
| --- | --- |
| **WWMP** | 1 ± 0.27 a |
| **DSMP** | 1.07 ± 0.43 a |

WWMP = Well-watered mycorrhizal plant; DSMP = Drought-stress mycorrhizal plant.
